# The Peritoneum: Beyond the Tissue – A Review

**DOI:** 10.3389/fphys.2018.00738

**Published:** 2018-06-15

**Authors:** Andres Isaza-Restrepo, Juan S. Martin-Saavedra, Juan L. Velez-Leal, Felipe Vargas-Barato, Rafael Riveros-Dueñas

**Affiliations:** ^1^Escuela de Medicina y Ciencias de la Salud, Universidad del Rosario, Bogotá, Colombia; ^2^Department of Clinical Surgery, Hospital Universitario Mayor – Méderi, Bogotá, Colombia; ^3^Clinical Research Group, Escuela de Medicina y Ciencias de la Salud, Universidad del Rosario, Bogotá, Colombia; ^4^Escuela de Medicina y Ciencias de la Salud, Universidad del Rosario, Bogotá, Colombia

**Keywords:** peritoneum, mesothelial cell, lymphatic stomata, anatomy, ultrastructure, embryogenesis

## Abstract

**Background:** Despite its complexity, the peritoneum is usually underestimated in classical medical texts simply as the surrounding tissue (serous membrane) of the gut. Novel findings on physiology and morphology of the peritoneum and mesothelial cell exist but they are usually focused or limited to Continuous Ambulatory Peritoneal Dialysis research and practice. This review aims to expose, describe and analyze the most recent evidence on the peritoneum’s morphology, embryology and physiology.

**Materials and Methods:** A literature review was performed on Pubmed and MEDLINE. With no limit of publication date, original papers and literature reviews about the peritoneum, the peritoneal cavity, peritoneal fluid, and mesothelial cells were included (*n* = 72).

**Results:** Peritoneum develops in close relationship to the gut from an early period in embryogenesis. Analyzing together the development of the primitive gut and the surrounding mesothelium helps understanding that the peritoneal cavity, the mesenteries and other structures can be considered parts of the peritoneum. However, some authors consider that structures like the mesenteries are different to the peritoneum. The mesothelial cell has a complex ultrastructural organization with intercellular junctions and apical microvilli. This complexity is further proven by the large array of functions like selective fluid and cell transport; physiological protective barrier; immune induction, modulation, and inhibition; tissue repair and scarring; preventing adhesion and tumoral dissemination; cellular migration; and the epithelial-mesenchymal transition capacity.

**Conclusion:** Recent evidence on the anatomy, histology, and physiology of the peritoneum, shows that this structure is more complex than a simple serous membrane. These results call for a new conceptualization of peritoneum, and highlight the need of adequate research for identifying clinical relevance of this knowledge.

## Introduction

A large amount of everyday surgeries occur in the peritoneal cavity (Kingsnorth and LeBlanc, 2003; Matthews and Neumayer, 2008), and in clinical practice surgeons and all type of physicians are in constant look for signs of peritoneal irritation. Nonetheless, peritoneal cavity is usually disregarded as an empty space without clear clinical significance (Sasaki, 1999) and the peritoneum simply as the covering tissue (serous membrane) of abdominal viscera in classic physiology (Hall and Guyton, 2015; Boron and Boulpaep, 2017), and histology texts (Ross and Pawlina, 2012).

This perspective of the peritoneum is a result of the classic “anatomical view” (Canogar, 2004), usually considered as reductionist. Another perspective is the “systemic view” proposed by von Bertalanffy (1968), which is centered in the complex interactions of molecules, cells, tissues, and organs that may be more appropriate for the comprehension of the peritoneum.

De Lamballe in 1829, was the first to highlight the protective functions of the peritoneum on the abdominal cavity, and in 1880 Senn, based on this knowledge, used omental flaps to protect intestinal sutures (Liebermann-Meffert, 2000). Recently, discussions on the anatomical concepts around the mesenteries have emerged. Interestingly, this new appraisal recognizes that the mesentery is composed of peritoneum, but is presented as an independent structure (Culligan et al., 2013; Sehgal and Coffey, 2014; Coffey and O’Leary, 2016).

Recent literature shows that peritoneal research is becoming a dynamic field where complex relationships have been described. Nonetheless, this research has been focused on Continuous Ambulatory Peritoneal Dialysis when it could be used in other clinical context and research. To better understand the complex relationships of the peritoneum, a literature review on the anatomy, embryology and physiology of the peritoneum and mesothelial cell was conducted.

## Materials and Methods

A comprehensive search on Pubmed and MEDLINE was performed using the following Mesh terms: peritoneum, mesothelium, immunity, peritoneal cavity, scarring, embryogenesis, lymphatic stomata, anatomy, and ultrastructure. Additional non-mesh terms were used: antimicrobial peptides, adhesion molecules, chemokines, and peritoneal fluid. Original studies and reviews assessing any of the following topics were included: embryologic development of the primitive gut or peritoneum; anatomy or morphological organization of the peritoneum or peritoneal cavity; histology or ultrastructural organization of mesothelial cell; functions or physiologic properties of mesothelial cells.

All papers published at any time or any language were included. Original studies focused only on mesothelial cell from the pleura or pericardium were excluded. A total of 48 original papers and 24 reviews (see **Table 1**) were included.

**Table 1:** Included articles.

Type of article	Total of articles included	Reference
Original studies	48	Tsilibary and Wissig, 1983; Davies et al., 1990; Van Hinsbergh et al., 1990; Van Vugt et al., 1991, 1992, 1996; Jonjic et al., 1992; Pronk et al., 1992; Marshall et al., 1993; Abu-Hijleh et al., 1995; Valle et al., 1995; Liberek et al., 1996; Mutsaers et al., 1996; Li et al., 1997; Wassilev et al., 1998; Chunfeng et al., 1999; Michailova et al., 1999; Sasaki, 1999; Hausmann et al., 2000; Liang and Sasaki, 2000; Michailova, 2001; Zarrinkalam et al., 2001; Bellingan et al., 2002; Boulanger et al., 2002; Cui et al., 2002; Kluth et al., 2003; Bird et al., 2004; Kato et al., 2004; Tang et al., 2004; Yao et al., 2004; Saed et al., 2006; Grupp et al., 2007; Park et al., 2007; Krediet et al., 2008; Yamaji et al., 2008; Rangel-Moreno et al., 2009; Colmont et al., 2011; Katz et al., 2011; Culligan et al., 2012, 2013, 2014; Wang et al., 2012; Wang J. et al., 2013; Wang J.X. et al., 2013; Yuchang et al., 2013; Retana et al., 2015; Coffey et al., 2016; Hwang et al., 2016; Shaw et al., 2016
Reviews	24	Minot, 1890; Heel and Hall, 1996; Healy and Reznek, 1998; Liebermann-Meffert, 2000; Mutsaers, 2002; Herrick and Mutsaers, 2004; Mutsaers, 2004; Glik and Douvdevani, 2006; McCully and Madrenas, 2006; Yung et al., 2006; Bricou et al., 2008, 2009; Burn and Hill, 2009; Kazancioglu, 2009; Devuyst et al., 2010; Wang et al., 2010; Patel and Planche, 2012; Susan and Tak Mao, 2012; Tirkes et al., 2012; Culligan et al., 2013; Blackburn and Stanton, 2014; Sehgal and Coffey, 2014; Coffey and O’Leary, 2016; van Baal et al., 2017

## Results

### Embryogenesis and Anatomy

The peritoneum is part of the abdominal cavity and the largest of the three serosal cavities of the human body. Serosal cavities were described by Bichart in 1827 (Herrick and Mutsaers, 2004; Mutsaers, 2004), and Minot (1890) described two mesodermal tissues: the *mesothelium* as the epithelial lining of the embryonic serosal cavity (caelom), and *mesenchyme* as the non-epithelial mesoderm (Minot, 1890). The anatomic organization of the abdominal cavity is consequence of the complex embryologic development of the gut and the peritoneum (Coffey and O’Leary, 2016; Brenkman et al., 2017).

**FIGURE 1:**
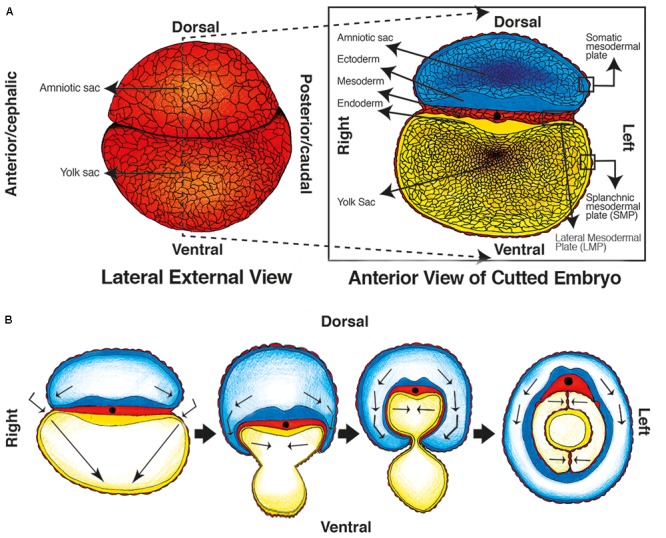
Embryologic development of the primitive gut, primitive mesenteries and Coelomic cavity. **(A)** Week 5: Lateral view of the external embryo covered by mesoderm and separated in two sacs/cavities (left image), and an anterior view of a transverse cut of the embryo, the gastrulation has ended and the three layered disk is visible between the cavities. **(B)** Weeks 5–7: transverse folding of the embryo occur, the caelom cavity and primitive gut are formed, and the amniotic cavity covers the embryo.

Peritoneum starts developing during the gastrulation process (van Baal et al., 2017), alongside the primitive gut (Burn and Hill, 2009; Tirkes et al., 2012; Blackburn and Stanton, 2014; Coffey and O’Leary, 2016). During the 1st weeks of development, a three-layer flat disk is formed and separates the amniotic cavity and yolk sac. It is composed of ectoderm, endoderm, and the mesoderm in-between (van Baal et al., 2017). The mesoderm differentiates into paraxial (surrounding the neural tube), intermediate, and lateral mesodermal plate (LMP). The LMP continues out of the flat disk and covers the ectoderm of the amniotic cavity (somatic mesodermal plate) (Herrick and Mutsaers, 2004; van Baal et al., 2017), and the endoderm of the yolk sac [splanchnic mesodermal plate (SMP)] (see **Figure 1A**) (Herrick and Mutsaers, 2004; Burn and Hill, 2009; van Baal et al., 2017).

Later on, the “flat disk” curves transversally (Burn and Hill, 2009; Blackburn and Stanton, 2014; van Baal et al., 2017), and the amniotic cavity “hugs” the embryo until the endodermic tube closes and forms the primitive gut. Consequently, the amniotic cavity surrounds the body of the embryo; the yolk sac disappears; and the primitive gut is located in the midline of the anterior-posterior axis inside the new coelomic cavity (Herrick and Mutsaers, 2004; van Baal et al., 2017) (see **Figure 1B**).

The coelomic cavity will be composed of a mesothelial layer that covers the primitive gut (formed from the SMP), a second layer that covers the cavity’s wall (derived from the somatic plate), and the space in-between. The layer covering the gut will be known later as the visceral peritoneum and the one covering the wall will be known as the parietal peritoneum (Herrick and Mutsaers, 2004; van Baal et al., 2017). The closing process of the primitive gut brings together two opposing layers of mesothelium (peritoneum) ventrally and dorsally to the gut, which are known as primitive mesenteries (Patel and Planche, 2012; Tirkes et al., 2012) (**Figure 1B**). This embryologic process proves that the mesenteries are a peritoneal derived structure.

**FIGURE 2:**
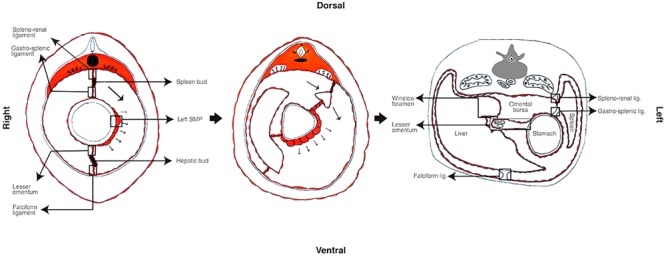
Primitive mesentery differentiation. In the rat, the left–right asymmetry begins approximately at embryologic 9.5 days of development. The left SMP differentiates and interacts with the foregut that outgrows to the left, the spleen bud begins differentiating and moves to left pushing the liver in formation to the right. During this period the primitive mesenteries differentiate to hepatic and spleen buds, ligaments and the lesser omentum.

The primitive gut divides into fore, mid and hindgut (Kluth et al., 2003; Blackburn and Stanton, 2014), and simultaneous differentiation processes occur at each level. While the gut differentiates, the mesenteries and the covering peritoneum also develop (Patel and Planche, 2012; Tirkes et al., 2012; Blackburn and Stanton, 2014). At the level of the foregut, a hepatic and splenic bud is formed from the ventral and dorsal mesenteries, respectively (Patel and Planche, 2012; Tirkes et al., 2012; Blackburn and Stanton, 2014). The dorsal mesentery connected directly to the spleen will become the spleno-renal ligament; and the remnant between the spleen and the stomach will be the gastro-splenic ligament (Patel and Planche, 2012; Blackburn and Stanton, 2014). The ventral mesentery between the forming liver and cavity wall will turn into the falciform ligament; and the one between the liver and the stomach will be the lesser omentum that contains the biliary tract, hepatic artery, and portal vein (Patel and Planche, 2012; Tirkes et al., 2012; Blackburn and Stanton, 2014) (see **Figure 2**). In consequence the ligaments and the lesser omentum can also be considered peritoneal derived structures.

The midgut development is classically described as a 270° counterclockwise rotation (Kluth et al., 2003; Blackburn and Stanton, 2014; Coffey and O’Leary, 2016), but an observational study suggested that there was no such rotation (Kluth et al., 2003). Initially, a duodenum loop is formed and lengthens inside the cavity pushing the small intestines and the caecum outside of the embryo (physiological herniation) (Kluth et al., 2003; Blackburn and Stanton, 2014). Later on, the small intestines, the terminal ileum, and caecum enter back (Kluth et al., 2003). When the caecum is returning, the digestive tract has grown to the left (Burn and Hill, 2009), so it has to locate at lower right quadrant (Kluth et al., 2003).

This observations explains why some parts of the digestive tract remain suspended to a stretched mesentery (small intestine, transverse colon and sigmoid) while others adheres to the posterior wall (duodenum, caecum, right and left colon) (Blackburn and Stanton, 2014; Coffey and O’Leary, 2016), which has been traditionally described as a regression of he mesenteries (Tirkes et al., 2012; Blackburn and Stanton, 2014; Coffey and O’Leary, 2016). Coffey et al. suggests that rather than a regression a flattening occurs, and that the mesenteries are contiguous in both fetal and adult life (Coffey and O’Leary, 2016; Coffey et al., 2016). This contiguity was observed in anatomical studies of cadaveric bodies (Culligan et al., 2012), and further proved by histological analysis of the mesenteries (Culligan et al., 2014). Interestingly, histological findings by Culligan et al. (2014) not only prove the adult contiguity of the mesentery, but show that it is composed of peritoneal mesothelium.

Another important step in the embryogenesis of the digestive tract and peritoneum is the left–right asymmetry (Burn and Hill, 2009). The mesothelium of the left SMP thickens and induces a rapid grow of the left side of the foregut (see **Figure 2**) (Burn and Hill, 2009), which explains the larger stomach’s left curve (Liebermann-Meffert, 2000; Burn and Hill, 2009), the movement of the spleen to the left side and the liver to the right (Burn and Hill, 2009; Patel and Planche, 2012; Tirkes et al., 2012; Blackburn and Stanton, 2014). This left SMP specialization induces a thickening of the left side of the dorsal mesentery, resulting in a left tilting of the mesenteries and gut (Burn and Hill, 2009). While the left side of the stomach outgrows, mesothelial tissue sprouts from the curvature until it fuses to the transverse colon forming the greater omentum (Liebermann-Meffert, 2000).

**FIGURE 3:**
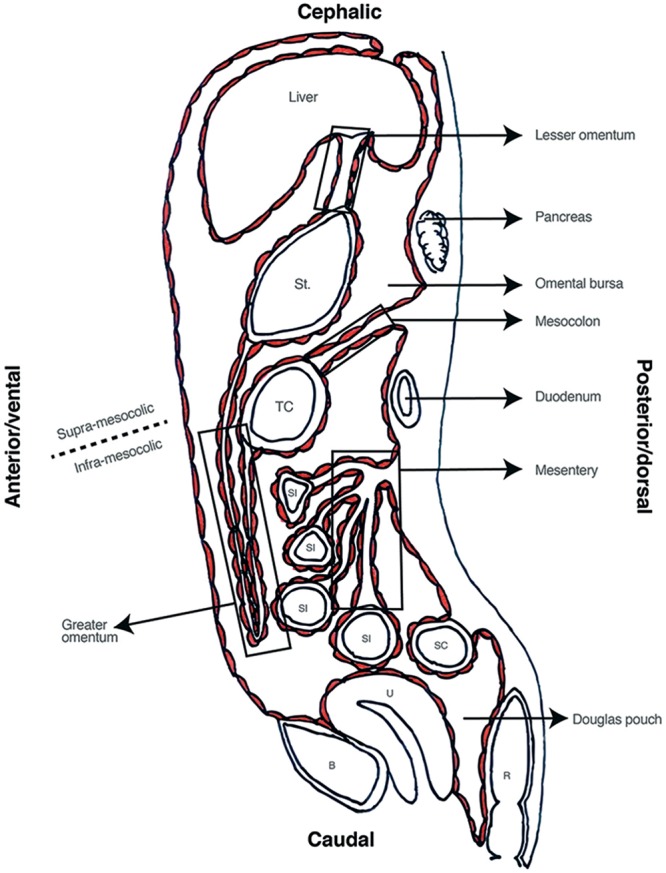
Sagittal view of abdominal cavity. St, stomach; TC, transverse colon; SI, small intestine; SC, sigmoid colon; R, Rectum; U, Uterus; B, bladder.

The final product is a left sided digestive tract (Burn and Hill, 2009), covered by visceral peritoneum, a parietal layer adjacent to abdominal wall, and the liquid-filled space in between (Healy and Reznek, 1998; Herrick and Mutsaers, 2004; Mutsaers, 2004; Patel and Planche, 2012; Tirkes et al., 2012; Blackburn and Stanton, 2014; van Baal et al., 2017). Embryologic peritoneum fuses forming the primitive mesenteries that give rise to supporting ligaments (e.g., falciform ligament) (Healy and Reznek, 1998; Patel and Planche, 2012; Tirkes et al., 2012; Blackburn and Stanton, 2014); the lesser and greater omentum (Healy and Reznek, 1998; Liebermann-Meffert, 2000; Patel and Planche, 2012; Tirkes et al., 2012; Blackburn and Stanton, 2014); and the adult mesentery (Culligan et al., 2012, 2014; Coffey and O’Leary, 2016; Coffey et al., 2016). Inferiorly, the peritoneum forms the roof of the pelvic cavity and in women forms the Douglas pouch between the uterus and rectum (Blackburn and Stanton, 2014) (see **Figure 3**).

**FIGURE 4:**
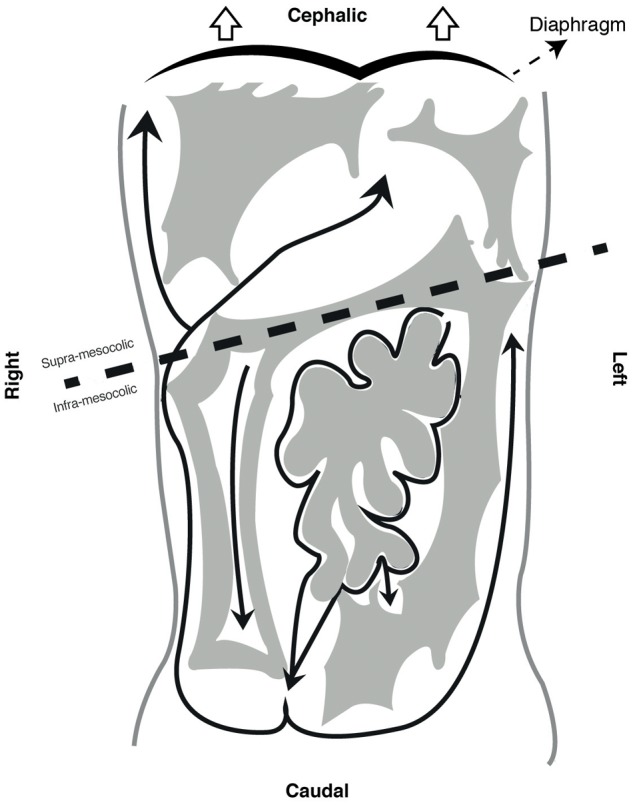
Peritoneal compartments and peritoneal fluid flow.

This final anatomical distribution allows dividing the cavity in several spaces. Transverse mesocolon separates the cavity in supra and sub-mesocolic compartments which are then divided in subspaces (Healy and Reznek, 1998; Tirkes et al., 2012). An example of this is the omental bursa, the space formed from the left movement of the abdominal organs during embryogenesis, which is limited anteriorly by the lesser omentum and stomach’s posterior wall (see **Figure 3**). Access to this space is of great importance for lymphadenectomy and bursectomy in gastric cancer surgical treatment (Kayaalp, 2015; Brenkman et al., 2017). The fact that the digestive tube and the peritoneum are formed as contiguous structures, explains why all these subspaces are connected (e.g., Winslow foramen) allowing free peritoneal fluid flow through the entire cavity (Bricou et al., 2008, 2009) (see **Figure 4**).

### Histology and Physiology of the Mesothelial Cell

**FIGURE 5:**
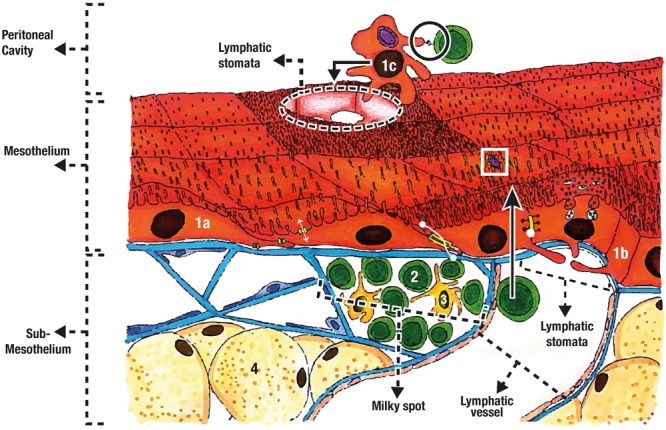
Mesothelial organization and functions. The mesothelium is composed of flat mesothelial cells (1a), and cuboidal mesothelial cells (1b). Water transport (two headed white arrow) occurs through aquaporins, while zonula adherens (two headed dot arrow) and tight junctions (white dot) give support and selective barrier properties. Mesothelial cell can also trap pathogens (white square), detach (1c), phagocyte pathogens and present antigen (black circle) for immune induction. The sub-mesothelium contains the basal membrane, the connective tissue, adipocytes (4) and the milky spots were mainly lymphocytes (2) and macrophages are found (3).

The peritoneum is generally described as a protective barrier and frictionless interphase that covers abdominal viscera (Herrick and Mutsaers, 2004; Mutsaers, 2004; Yung et al., 2006; Susan and Tak Mao, 2012), but it is a much more complex structure with a great variety of functions. Besides from participating in the embryogenesis of primitive gut (Burn and Hill, 2009), peritoneal functions include: selective fluid and cell transport (Mutsaers, 2002, 2004; Susan and Tak Mao, 2012; Retana et al., 2015; van Baal et al., 2017); physiological barrier (Davies et al., 1990; Heel and Hall, 1996; Zarrinkalam et al., 2001; Grupp et al., 2007; Kazancioglu, 2009; Susan and Tak Mao, 2012); immune induction, modulation, and inhibition (Bird et al., 2004; Susan and Tak Mao, 2012; van Baal et al., 2017); tissue repair and scarring (Susan and Tak Mao, 2012; van Baal et al., 2017); preventing adhesion and tumoral dissemination (Mutsaers, 2002, 2004); and *trans*-cellular migration (see **Figure 5**) (Mutsaers, 2002; Herrick and Mutsaers, 2004; Yung et al., 2006; Wang et al., 2010; van Baal et al., 2017).

Two mesothelial layers (visceral and parietal peritoneum) and the liquid-filled space in-between compose the peritoneal cavity (Herrick and Mutsaers, 2004; Mutsaers, 2004; van Baal et al., 2017). Each mesothelium is a tortuous monolayer of overlapped mesothelial cells (Mutsaers, 2002, 2004; Retana et al., 2015) that rests on a basal lamina and its connective tissue underneath (sub-mesothelium) (Michailova et al., 1999; Mutsaers, 2002; Bird et al., 2004; Yung et al., 2006; Blackburn and Stanton, 2014; Retana et al., 2015; van Baal et al., 2017). Sub-mesothelium varies throughout the peritoneum (van Baal et al., 2017), but it invariably contains different cell types (fibroblast, adipocytes, and others), and blood and lymph vessels (Bird et al., 2004).

#### Ultrastructural Features of the Mesothelial Cell

Mesothelial cell have cellular unions like tight junctions (zonula occludens), intermediate junctions (zonula adherens), desmosomes and gap junctions (Mutsaers, 2002, 2004; Susan and Tak Mao, 2012; Blackburn and Stanton, 2014; van Baal et al., 2017). Tight junctions provide semipermeable properties and work as a gate regulator for water, ions, and other solutes diffusion (Retana et al., 2015). Zonula adherens give structural support (Mutsaers, 2004), while gap junctions are mainly aqueous intracellular channels that alongside the lymphatic stomata and intermediate pores, compose the three-pore theory (Mutsaers, 2002, 2004; Krediet et al., 2008; Devuyst et al., 2010). This theory explains the ultra-filtrating properties of the peritoneum used for dialysis.

There are two main types of mesothelial cells: flattened epithelial-like and cuboidal (Abu-Hijleh et al., 1995; Michailova et al., 1999; Mutsaers, 2002, 2004; Bird et al., 2004; Yung et al., 2006), and some describe an intermediate type (Michailova et al., 1999; van Baal et al., 2017). The type of cell varies depending on anatomic relationships. For example, cuboidal type are found near parenchymal viscera and near lymphatic stomata (see **Figure 5**), while flattened type is more common in intestinal, parietal, omental (Mutsaers, 2002, 2004; van Baal et al., 2017), and mesenteric mesothelium (Culligan et al., 2014).

All mesothelial cells have apical microvilli (Mutsaers et al., 1996; Michailova et al., 1999; Mutsaers, 2002, 2004; Yung et al., 2006; Susan and Tak Mao, 2012; van Baal et al., 2017), but their density changes depending on peritoneal location (Mutsaers et al., 1996; Michailova et al., 1999; van Baal et al., 2017). Microvilli density and distribution also changes with physiologic or pathologic states (Mutsaers et al., 1996; Susan and Tak Mao, 2012). Mutsaers et al. (1996) found that after injury the mesothelial cells surrounding the wound become cuboidal and the density of microvilli increases. It has been suggested that microvilli can capture molecules and serous exudates (Mutsaers et al., 1996; Mutsaers, 2002; Susan and Tak Mao, 2012), bacteria (see white square at **Figure 5**) (Wang J. et al., 2013), and leukocyte (Mutsaers et al., 1996; Liang and Sasaki, 2000; Bellingan et al., 2002; Mutsaers, 2002; Susan and Tak Mao, 2012), contributing to its barrier functions.

These barrier functions make the peritoneum the first line of defense of the abdominal cavity (Susan and Tak Mao, 2012). For example, peritoneal dialysis has been found to alter the cellular junctions of mesothelial cells (Retana et al., 2015). Therefore, in peritoneal dialysis patients, the barrier functions are altered, which explains the increased risk for peritonitis (Susan and Tak Mao, 2012). During surgery, the peritoneum is injured and the barrier interrupted, so is fair to assume that the larger the injury, the higher the risk of infection. This is supported by the increasing tendency for minimally invasive surgery (Reim et al., 2015).

Moreover, anionic sites at the glycocalyx (Mutsaers et al., 1996) and adhesion molecules in microvilli of the mesothelial cell, change in response to pathogenic stimuli (Jonjic et al., 1992; Liberek et al., 1996; Liang and Sasaki, 2000; Michailova, 2001; Bellingan et al., 2002; Herrick and Mutsaers, 2004; Susan and Tak Mao, 2012). The main adhesion molecules found in mesothelial cells are ICAM-1 and VCAM-1 (Jonjic et al., 1992; Liberek et al., 1996; Liang and Sasaki, 2000; Boulanger et al., 2002; Cui et al., 2002), both increase expression when stimulated with pathogen related molecules (Liang and Sasaki, 2000), INF-γ, (Valle et al., 1995; Hausmann et al., 2000; Shaw et al., 2016) Il-1β, and TNF-α (Jonjic et al., 1992; Liberek et al., 1996). These changes in adhesion molecules regulate leukocyte migration (Jonjic et al., 1992; Liberek et al., 1996; Bellingan et al., 2002) and autophagy-mediated bacterial removal (Wang J. et al., 2013).

#### Cytokine Production and Pathogen Recognition

Mesothelial cell has demonstrated immunomodulatory functions through the expression of cytokines like MCP-1 (CCL2) (Jonjic et al., 1992; Kato et al., 2004; Park et al., 2007), MIP-2 (Kato et al., 2004), CXCL1 (Park et al., 2007), Il-6 (Yao et al., 2004; Yamaji et al., 2008), TNF-α, Il-1β (Yao et al., 2004), Il-8 (CXCL8) (Jonjic et al., 1992; Colmont et al., 2011), Il-10 (Yao et al., 2004), and Il-15 (Hausmann et al., 2000). The production of these molecules is complemented by the fact that mesothelial cell recognizes inflammatory processes.

Kato et al. (2004) demonstrated constituent expression of Toll-like receptors (TLR) 1-6, CD14, and MD-2 (required for TLR-4 signal transduction) (Kato et al., 2004). Other authors have reached similar results, proving expression of TLR-1 (Colmont et al., 2011), TLR-3 and TLR-2 (Park et al., 2007; Colmont et al., 2011; Hwang et al., 2016), TLR-4 (Park et al., 2007; Colmont et al., 2011; Wang J. et al., 2013; Hwang et al., 2016), TLR-6 (Colmont et al., 2011), and TLR-5 (Park et al., 2007; Colmont et al., 2011). Other receptors like nucleotide-binding oligomerization domain (Nod)-1 and Nod-2 (Park et al., 2007), and AGE receptors (RAGE) (Boulanger et al., 2002) have been identified. Some of these studies were done on murine or mice models (Kato et al., 2004; Park et al., 2007; Hwang et al., 2016) while others were done on human peritoneal mesothelial cells (Boulanger et al., 2002; Colmont et al., 2011; Wang J. et al., 2013). All these are pathogen, or injury related, recognition receptors, which are an important component of innate immunity (Turvey and Broide, 2010).

#### Cellular Transmigration, Immune Induction and Antigen Presentation

Lymphatic stomata are located near the milky spots and were described by Von Recklinghausen in 1863 (Van Vugt et al., 1996; Wang et al., 2010). They are highly important for leukocyte migration, inflammatory response, and fluid drainage from the peritoneum (Abu-Hijleh et al., 1995; Wang et al., 2010). Stomata are “pores” formed between cuboidal mesothelial cells. This cells have cytoplasmic processes that extend to the interior of the pore (see **Figure 5**) (Abu-Hijleh et al., 1995; Wassilev et al., 1998; Cui et al., 2002; Wang et al., 2010). These cytoplasmic processes may act as “closing doors” for either drainage or inflow to the peritoneal cavity. This was suggested by the identification of contractile filaments on the cells surrounding the stomata (Tsilibary and Wissig, 1983; Abu-Hijleh et al., 1995; Heel and Hall, 1996; Wang et al., 2010). Moreover, it has been observed that stomata change in size and number after bacterial injection (Michailova, 2001), and 6 min later, about 50% will be drained into the thoracic tube (Heel and Hall, 1996; Kazancioglu, 2009).

Milky-spots are specialized tissue with abundant populations of leukocytes that are found mainly in the greater omentum (see **Figure 5**) (Van Vugt et al., 1996; Cui et al., 2002; Glik and Douvdevani, 2006; Rangel-Moreno et al., 2009). These spots exhibit many lymphoid associated tissue properties (Rangel-Moreno et al., 2009), and are essential in T-cell response (Glik and Douvdevani, 2006; Rangel-Moreno et al., 2009). In the absence of spleen and Peyer patches, mesothelial cells were found to migrate to the greater omentum’s milky spots where production of IgG and IgM, formation of B-cell germinal centers, and T-cell response, was observed (Rangel-Moreno et al., 2009).

As mentioned before, many authors have demonstrated ICAM-1 and VCAM-1 expression especially in mesothelial microvilli (Jonjic et al., 1992; Liberek et al., 1996; Liang and Sasaki, 2000; Boulanger et al., 2002; Cui et al., 2002). Interestingly, macrophages and lymphocytes of milky-spots express correspondent adhesion molecules (Cui et al., 2002). Cui et al. (2002) observed that mesothelial cell near the spots had higher expression of adhesion molecules. Supporting this, Bellingan et al. (2002) identified that adhesion molecules regulated macrophage clearance. Therefore, peritoneum not only drains the cavity but also mediates its clearance.

Antigen presentation is an important part for T-cell and B-cell immune responses (Glik and Douvdevani, 2006; McCully and Madrenas, 2006; Turvey and Broide, 2010). It occurs in the milky-spots despite the lack of follicular and inter-digitating dendritic cell networks (Van Vugt et al., 1996; Rangel-Moreno et al., 2009). Dendritic cells have been observed in milky spots only after bacterial immunization (Van Vugt et al., 1996), while in peritoneal cavity they can be seen during steady state (Van Vugt et al., 1991), but are increased after bacterial immunization (Van Vugt et al., 1992). These findings suggest that other cells, different from macrophages and dendritic cells, may have antigen presentation (AP) functions.

Several findings suggest a possible AP function by the mesothelial cells (Valle et al., 1995; Hausmann et al., 2000; Shaw et al., 2016). Major Histocompatibility Complex class II (MHC-II) is expressed by mesothelial cell in steady state (Valle et al., 1995) and after IFN-γ stimulation (Valle et al., 1995; Hausmann et al., 2000; Shaw et al., 2016). Accessory MHC-II molecules like ICAM-1 (Jonjic et al., 1992; Liberek et al., 1996; Hausmann et al., 2000; Liang and Sasaki, 2000; Boulanger et al., 2002; Cui et al., 2002; Shaw et al., 2016), LFA-1 and low levels of B7-1 (Shaw et al., 2016), have are also expressed by mesothelial cell. Moreover, pure T-cell proliferation induction (Valle et al., 1995; Hausmann et al., 2000; Shaw et al., 2016), and phagocytic activity by mesothelial cells have also been reported (Valle et al., 1995; Hausmann et al., 2000; Wang J. et al., 2013; Shaw et al., 2016).

Lymphatic stomata and the milky-spots are specialized structures fundamental in cleaning the cavity during an inflammatory process. Stomata seem to serve as a physical protective mechanism through the drainage of bacteria and inflammatory residues, while milky-spots serve as specialized tissue for regulation of the inflammatory response and elimination of the inflammatory agent. The mesothelial cell through cytokine production, antigen presentation, and phagocytic functions, are the regulators of this complex interaction of the peritoneum and the immune system. In consequence, surgeons should think twice before removing the greater omentum (Van Vugt et al., 1996; Cui et al., 2002; Glik and Douvdevani, 2006; Rangel-Moreno et al., 2009), and more studies are required to evaluate the effects of removing, partially or totally, this structure.

#### Tissue Repair and Scarring

Inadequate resolution of an inflammatory response leads to persistent macrophage activity and tissue destruction (Bellingan et al., 2002; Susan and Tak Mao, 2012). Mesothelial cells regulates macrophage clearance (Bellingan et al., 2002), while also producing matrix metalloproteinase (MMP) activators and inhibitors for tissue repair and scarring (Marshall et al., 1993; Chunfeng et al., 1999; Saed et al., 2006).

**FIGURE 6:**
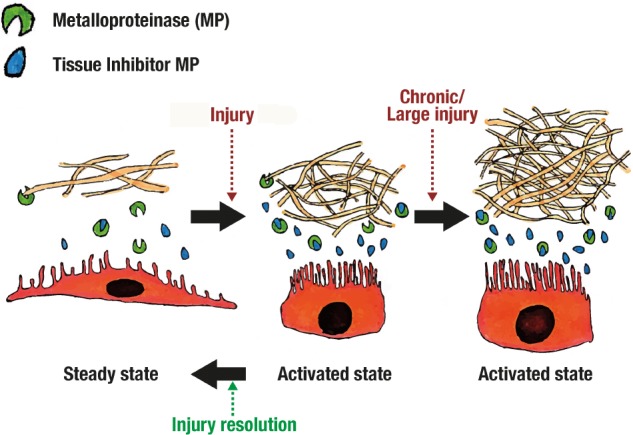
Adherence formation.

As mentioned before, mesothelial cells (especially those near milky spots) change its phenotype in response to injury (Tsilibary and Wissig, 1983; Mutsaers et al., 1996), and it returns to normality only after tissue repair (see **Figure 6**). This mesothelial change might be an active form that participates through the complete inflammatory process, including tissue repair. Additionally, mesothelial cells have demonstrated the ability to participate in fibrinolytic (Van Hinsbergh et al., 1990), procoagulant (Pronk et al., 1992), and fibrinogenic activity (Davies et al., 1990).

Repair and scarring is complex and dynamic. Mesothelial cell in steady state produces Tissue Inhibitors of metalloproteinase (TIMP), 72 and 92-kD gelatinase and little to none collagenase (MMP1) (Marshall et al., 1993; Chunfeng et al., 1999). When stimulated with phorbol myristate acetate (PMA), TIMP, 92-kD gelatinase and collagenase production is enhanced (Marshall et al., 1993). It seems that, in steady state, extracellular matrix degradation is balanced but easily enhanced through certain inflammatory pathways (see **Figure 6**). This is supported by the fact that after injury mesothelial cells separate from each other (Van Hinsbergh et al., 1990; Chunfeng et al., 1999; Retana et al., 2015), and expose extracellular matrix components like collagen I and III (Pronk et al., 1992).

Scarring processes may differ by the type of injury. For example, TIMP and collagenase production was enhanced by TNF-α (Marshall et al., 1993), and transformation growth factor beta (TGF-β) (Chunfeng et al., 1999). Only TIMP was enhanced with Il-1β but the greatest enhancement was achieved using both TNF-α and Il-1β (Marshall et al., 1993). On the other hand, stimulation with Tisseel (composed of fibrinogen, thrombin, aprotinin and CaCl_2_) enhances production of MMP1 and 2 while reducing TIMP1 (Saed et al., 2006). This explains why adhesions are formed and sometimes is protective and others a complication (see **Figure 6**). Understanding these functions and interactions in scarring and repair should help elucidate and develop new approaches for avoiding surgery related complications, or even use mesothelium for tissue engineering and repair.

Some authors have observed that mesothelial cells can detach and differentiate to hepatic stellate cells (HSC), myofibroblast (Yuchang et al., 2013), and macrophage-like cells (Katz et al., 2011). This process is known as epithelial mesenchymal transition (Herrick and Mutsaers, 2004; Katz et al., 2011; Yuchang et al., 2013). Further studies are needed for understanding this mesothelial function, but this advocates that mesothelial cell may have more complex functions related to tissue repair.

### Peritoneal Fluid Physiology and Drainage

The peritoneal fluid separates both layers of mesothelium with a quantity of 5–100 ml in volume (Blackburn and Stanton, 2014; van Baal et al., 2017). It is described as an ultra-filtrated blood derivate (Heel and Hall, 1996; Blackburn and Stanton, 2014), containing immune elements like complement’s C3, C4 (Heel and Hall, 1996; Tang et al., 2004), and immunoglobulin G (Davies et al., 1990); antimicrobial peptides like Human neutrophil peptide (HNP) 1 and 3, and Human β defensins (HβD) 1 to 3 (Zarrinkalam et al., 2001; Grupp et al., 2007); immune cells like macrophages, lymphocytes, eosinophils, mesothelial cells and mast cells (van Baal et al., 2017). All these humoral elements are produced by mesothelial cell (Zarrinkalam et al., 2001; Tang et al., 2004; Grupp et al., 2007) (see **Table 2**), and along with the cellular components, make the peritoneal fluid a physiological barrier against infection.

**Table 2:** Peritoneal fluid humoral components.

Component	Concentration	Detection
C3	4–6 μg/ml	Human peritoneal cell expression *in vitro* (Tang et al., 2004)
C4	0.5–1.6 μg/ml	Human peritoneal cell expression *in vitro* (Tang et al., 2004)
HNP1, 3	0.48 μg/ml	Human peritoneal cell expression *in vitro* (Grupp et al., 2007)
HβD 1	0.88 μg/ml	Human peritoneal cell expression *in vitro* (Grupp et al., 2007)
HβD 2	0.16–0.2^∗^ μg/ml	Human peritoneal cell expression *in vitro* (Grupp et al., 2007)
HβD 3	0.24 μg/ml	Human peritoneal cell expression *in vitro* (Grupp et al., 2007)
IgM	4^∗a^	Human peritoneal cell expression *in vitro* (Rangel-Moreno et al., 2009)
IgG	5^∗a^	Human peritoneal cell expression *in vitro* (Rangel-Moreno et al., 2009)

*^∗^Not expressed in steady state. ^*a*^Log10 titer.*

Peritoneal fluid can be considered a physiological barrier thanks to the presence of all these humoral and cellular components so maintenance of its composition should call our attention. Peritoneal dialysis solutions, ascites, and peritoneal lavage might change the fluid’s composition, but these should be answered through controlled and adequate conducted research.

Lymphatic stomata are the main structures responsible for peritoneal fluid drainage (Tsilibary and Wissig, 1983; Abu-Hijleh et al., 1995; Li et al., 1997; Wassilev et al., 1998; Bellingan et al., 2002; Wang et al., 2010; Wang J.X. et al., 2013), and are located mainly in diaphragmatic peritoneum. Stomata drain to lymphatic vessels (see **Figure 5**) (Abu-Hijleh et al., 1995; Li et al., 1997; Wang et al., 2010), passes through parasternal lymph nodes until it ends in the terminal thoracic duct (Abu-Hijleh et al., 1995). Diaphragmatic movement produces a change in hydrostatic pressure that moves peritoneal fluid upward (Abu-Hijleh et al., 1995; Bricou et al., 2008) (see **Figure 4**). Stomata’s have also been found in other serous membranes like tunica vaginalis (Wang et al., 2012; Wang J.X. et al., 2013), animal pericardium, and human pleura (Wang J.X. et al., 2013), suggesting a related lymphatic drainage system.

## Conclusion

Peritoneum and the digestive system are in constant interactions from early stages in embryogenesis. Analyzing development of both, shows that the mesenteries, supporting ligaments, lesser, and greater omentum are peritoneal derived structures. Complex relationships and functions of the peritoneum are more evident when its microscopic organization and physiology are studied. Further from its ultra-filtrating capacity, the peritoneal mesothelium is a physical barrier with the capacity to trap bacteria and molecules. Furthermore, drainage through the lymphatic stomata, and the presence of different humoral components in the peritoneal fluid, makes the peritoneal cavity a complex protective structure.

The mesothelial cell is capable of recognizing pathogen and tissue damage, and initiating inflammatory response through antigen presentation, cytokine production, interaction with immune cells like macrophages, and through tissue repair and adherence formation. Many questions on clinical practice arise when the peritoneum is studied on detail, but the lack of clinical evidence makes all conclusions and finding from this review to fall in purely theoretical appreciations. More clinical trials and observational research, studying the effects of peritoneal lavage, omental removal, or other practices are needed to identify clinical significance of the findings described on this review.

## Author Contributions

AI-R and JV-L contributed to conception and design of the work; literature search, data collection and analysis; drafting, writing, and critical review of the text. JM-S contributed to conception and design of the work; literature search, data collection and analysis; drafting, writing, and critical review of final document; figure design and drawing. FV-B contributed to conception and design of the work; literature search, data collection and analysis; drafting, and critical review of the text. RR-D contributed to conception and design of the work; literature search, data analysis; drafting, and critical review of the text.

## Conflict of Interest Statement

The authors declare that the research was conducted in the absence of any commercial or financial relationships that could be construed as a potential conflict of interest.
